# Anxiety-Related Attention Bias in Four- to Eight-Year-Olds: An Eye-Tracking Study

**DOI:** 10.3390/bs10120194

**Published:** 2020-12-17

**Authors:** Suzannah Stuijfzand, Bobby Stuijfzand, Shirley Reynolds, Helen Dodd

**Affiliations:** 1 School of Psychology and Clinical Language Sciences, University of Reading, Berkshire RG6 6AH, UK; suzannah.ravenscroft@chuv.ch (S.S.); s.a.reynolds@reading.ac.uk (S.R.); 2 Stuijfzand Data Consulting, 1000 Lausanne, Switzerland; bobby@stuijfzand-data.com

**Keywords:** anxiety, child, attention bias, eye tracking, gaze, development

## Abstract

(1) Background: There is evidence of an attention bias–anxiety relationship in children, but lack of appropriate methods has limited the number of studies with children younger than eight years old. This study used eye tracking as a measure of overt attention in young children. The aim of this study was to assess anxiety-related attention bias in children aged four to eight years. Age was considered a moderator, and the influence of effortful control was investigated. (2) Method: A community sample of 104 children was shown pairs of happy–neutral and angry–neutral faces. Growth curve analyses were used to examine patterns of gaze over time. (3) Results: Analyses revealed moderation by age and anxiety, with distinct patterns of anxiety-related biases seen in different age groups in the angry–neutral face trials. Effortful control did not account for age-related effects. (4) Conclusions: The results support a moderation model of the development of anxiety in children.

## 1. Introduction

Anxiety is one of the most prevalent mental health disorders in children and adolescents. Clinical anxiety affects an estimated 6.5% of children and adolescents worldwide at any one time [1], and it is estimated that before age twenty-one, 23.5% of youths have suffered from a clinically significant anxiety problem [2]. Given this, it is crucial to understand the mechanisms that cause and maintain anxiety in childhood.

Cognitive models of anxiety suggest that there are particular cognitive biases that predispose, cause and/or maintain anxiety (see [3] for an overview of these models). Attention, interpretation and memory biases are theorised to result from overactive threat-related schemas that guide the processing of incoming stimuli [4]. Cognitive models suggest that after an initial assessment for threat, anxious individuals have an attention bias whereby attention is disproportionately allocated to threat [3]. Two meta-analyses have found evidence of an anxiety-related attention bias to threat in adults [5] and children [5,6], though the size of the relationship appears to be smaller in children than in adolescents and adults [6].

Although there appears to be evidence of anxiety-related attention bias in children, the nature of the bias is not clear. There is evidence of attentional vigilance, with anxious children showing a greater attention bias towards threat than nonanxious children, e.g., [7,8,9]. However, there is also evidence that anxious children display attention bias away from threat [10,11].

The Vigilance-Avoidance hypothesis suggests that anxious individuals may show an initial vigilance towards threat, followed by avoidance of threat [12,13]. This may explain some of the inconsistency in findings if studies vary in when exactly the bias is assessed following the presentation of the relevant stimuli [12]. Evidence for a vigilance-avoidance pattern has been found in adults when the time course of attention was investigated [14,15] and in children with separation anxiety disorder [16].

A complicating factor in understanding the relationship between attention bias and anxiety in children is that this relationship may change with age. Field and Lester [17] suggested three models to describe how development in children may affect the attention bias–anxiety relationship. The ‘integral’ model sees attention biases as innate and only influenced by individual factors, i.e., anxiety. In this model, development has no influence, and we would not expect to see any changes in the relationship between anxiety and attention bias across age. In the ‘moderation‘ model, although biases may be present early in all children, they will only remain for those with particular individual factors (such as anxiety). Here we would therefore expect that the association between bias and anxiety varies across child age. Finally, the ‘acquisition‘ model proposes that the emergence of attention biases in children depends on the interaction of biases with social, emotional and cognitive development. In this final model, anxiety may drive or be the result of the appearance of biases; again, we would see changes in the relationship between anxiety and bias across age. The results of Field and Lester’s [17] review supported a moderation model. This model is further supported by the moderation by age effect found by Dudeney et al. [6], where the bias–anxiety association was stronger in adolescents than children.

Although some studies have found an influence of age on the presence or nature of the relationship between attention bias and anxiety [18,19], there are very few studies that assess the relationship between attention bias and anxiety in children younger than eight years. The average age in Dudeney et al.’s [6] meta-analysis was 11.98 years old, and only one study with children below age eight was included. The authors highlighted the lack of studies with young children. This means that although moderation by age was found, this cannot be reliably extrapolated to children under eight years. Thus, it is unclear if there is a relationship between attention bias and anxiety in young children. This is important for at least two reasons. First, from a theoretical perspective, to distinguish between the models described by Field and Lester [17], we need to know whether an attention bias is present in early childhood and if it is related to anxiety. Secondly, from a treatment perspective, Attention Bias Modification (ABM) procedures, e.g., [20,21], target attention biases to reduce anxiety. Understanding if an anxiety-related attention bias exists in younger children and the nature of this bias will indicate whether the assumptions behind these approaches are appropriate and if such approaches are appropriate for children experiencing elevated anxiety symptoms.

One developmental factor that is likely relevant for understanding potential changes in any relationship between bias and anxiety is effortful control. Effortful control includes inhibition and attention control, defined as the ability to inhibit a dominant response to perform a subdominant response to detect errors and engage in planning [22]. This skill first emerges in infancy [22] but shows considerable development between the ages of four and eight [23,24]. This has relevance for attention bias because the withdrawal of attention or avoidance of threat may depend upon voluntary top-down or effortful control [25]. Given that the ability to exert top-down control emerges with age, it therefore follows that the avoidance of threat, which is likely driven by more voluntary control processes, may be particularly sensitive to the developmental stage [21]. Indeed, developmental differences in the avoidance component to threat have been found [21]. This idea is somewhat in line with the inhibition hypothesis, which argues that it is the inability of children to inhibit their selective attention to threat that gives rise to the relationship between an attention bias and anxiety [26]. Kindt and colleagues [26] argue that all infants have a bias towards threat; however, as children’s top-down control of attention develops with age, most become able to inhibit this attention bias. Bias therefore only remains for children with poor inhibition, and this sustained attention to threat increases their anxiety. Evidence for this effect has been found [27], albeit inconsistently [28]. Thus, when considering the relationship between attention bias and anxiety in younger children, i.e., under eight years, their developing effortful control should be taken into account and may explain developmental differences in findings.

One likely reason for the paucity of studies examining the relationship between attention bias and anxiety in younger children is that the main experimental methods used to assess attention bias are not suitable for younger children. The most commonly used tasks for assessing attention bias are the dot-probe task and the emotional Stroop task (see [3] for a detailed description of these tasks). These tasks require participants to understand, remember and follow verbal instructions and maintain their concentration during the experiment. The measures of attentional bias rely on reaction time, and the tasks are sensitive to distraction, delay and motivation. These features call into question the appropriateness of such tasks for use with young children [29,30]. Beyond their appropriateness for children, both tasks have other significant limitations. For example, the dot-probe task only provides a single measure of attention and cannot capture shifts or changes in attention over time. It has also been argued that processes other than attentional bias may drive differences on the emotional Stroop task [31]; for example, anxious individuals may display behavioural freezing in the presence of threat, which operates independently of an attention bias [32].

In contrast to reaction time tasks, eye tracking provides a continuous measure of overt visual attention. Eye tracking can be used during a free-viewing paradigm, which simply requires children to look at images on a screen. The use of eye tracking therefore significantly reduces reliance on language and other cognitive abilities relative to traditional reaction time tasks [33]. Eye-tracking tasks have demonstrated attention biases to negative stimuli in children aged 4 to 10 [34,35] as well as differences in attention biases between adults and children. For example, in an eye-tracking study, negativity bias was found to decrease with age [35]. Furthermore, eye-tracking tasks have shown differences between anxious and nonanxious children in their attention towards angry faces, vigilance-avoidance patterns in anxious children [16] and avoidance of emotional faces in anxious young children [34]. Studies such as these show the potential of eye-tracking methodology to assess attention processes in children. However, only one of these studies included children four years and under [34], and none examined the influence of age or developmental factors such as effortful control on attention bias. Furthermore, none of these studies used data analysis techniques that could take full advantage of the continuous nature of eye-tracking data, such as growth curve analysis (GCA; [36]). GCA allows the assessment of individual differences in the time course of visual attention to emotional stimuli, for example, in [37,38].

This paper aims to establish if children aged four to eight years show an anxiety-related attention bias to emotional stimuli. It uses an eye-tracking task designed for children in which pairs of happy–neutral and angry–neutral faces are presented. Following the meta-analysis of Dudeney et al. [6] and evidence from recent eye-tracking studies, we hypothesised that all children will show an initial vigilance to emotional faces, which will be stronger for angry faces than happy faces. We further hypothesised that participants with higher levels of anxiety will show increased vigilance for angry faces followed by avoidance, relative to participants with lower levels of anxiety. Finally, we hypothesised that these anxiety-related effects would be moderated by age such that anxiety-related avoidance would be stronger in older than younger children. As theory and previous research suggest that effortful control may be a relevant developmental factor in the relationship between attention bias and anxiety, it was investigated whether any age-related effects remained after controlling for effortful control. Following criticisms of previous tasks assessing attention bias in children, the influence of verbal and nonverbal cognitive abilities on task performance are also investigated.

## 2. Materials and Methods

### 2.1. Participants

Participants were recruited via magazine advertisements, newsletters, local newspapers, posters in public places and leaflets handed out by local schools and at children’s groups. Parents of 351 children answered online screening questionnaires regarding their child’s anxiety using the Preschool Anxiety Scale (PAS; [39]) or the Spence Children’s Anxiety Scale (SCAS; [40]) depending on age (see below). Children identified by parents as having a diagnosis of Autism Spectrum disorder, Attention Deficit Hyperactivity Disorder or a Learning Disability were not invited to participate because these conditions have a propensity for anxiety and/or particular cognitive processing difficulties that could influence findings (*n* = 8). Children identified as having high anxiety (>1 SD above the normed mean as reported on https://www.scaswebsite.com) or low anxiety (below the normed mean as reported on https://www.scaswebsite.com) were invited to participate.

On the basis of a power calculation using the effect size from a meta-analysis of anxiety and attention bias and a straightforward high versus low group comparison, we initially aimed to recruit a sample of 134 participants (80% power, α = 0.05). Due to time constraints, one hundred and thirteen children were invited and completed the experimental tasks (65 males, *M_age_* = 6.06, *SD_age_* = 1.16, age range: 4.08- to 8.83-year-olds, 19 × 4-year-olds, 37 × 5-year-olds, 26 × 6-year-olds, 15 × 7-year-olds, 16 × 8-year-olds). Following data cleaning (see below), data from 104 children (62 males, *M_age_* = 6.02, *SD_age_* = 1.15, age range: 4 to 8 years) were used in the analysis. The final sample included 65 children with high anxiety and 39 children with low anxiety. The study therefore had 67% power to detect between-group differences in attention bias on the basis of an effect size of *d* = 0.49 (Bar-Haim et al.). The majority of parents were female (98.2%), identified their children’s ethnicity as being British (90%; 3% European; 3% Mixed (Arabic and white British); 3% Australian) and identified themselves as the primary caregiver (91%). Ethnicity information was only available for 39 children. However, this was missing due to a technical error so we can assume that the ethnicity reported in this subsample to be representative of the entire sample. The study took place in Berkshire, where the population is around 80% white and the remaining 20% represents a range of ethnicities.

### 2.2. Measures: Parents

Parents completed measures of anxiety, effortful control and autistic traits.

#### 2.2.1. Childhood Anxiety; Spence Child Anxiety Scale (SCAS) and Preschool Anxiety Scale (PAS) and Child Version; (SCAS)

Both anxiety measures yield a total score of general anxiety symptoms. In both measures, higher scores indicate higher anxiety. Parents of 4- to 6-year-olds completed the PAS, a 28-item questionnaire answered on a five-point Likert scale. The measure has strong psychometric properties aligned with DSM-IV diagnoses and good construct validity [39]. In this study, the total score of the PAS had excellent internal consistency (α = 0.93). Parents of the 7- and 8-year-old group answered the parallel measure, the SCAS, a 38-item questionnaire. The SCAS has shown good psychometric properties [39,40] and in this study had excellent internal consistency (α = 0.94).

#### 2.2.2. Child Behaviour Questionnaire—Effortful Control Scale (CBQ-EFC)

Following Eisenberg et al. [41], the Effortful Control scale was formed from five subscales from the Children’s Behaviour Questionnaire (CBQ; [42]): low-intensity pleasure, inhibitory control, perceptual sensitivity, attentional control and attention shifting. The CBQ assesses individual differences in attentional self-regulation as a basic dimension of temperament. Parents answered 52 items. Higher scores indicate more effortful control. Internal reliability in this study was excellent for the total Effortful Control scale (α = 0.88).

#### 2.2.3. The Autism Spectrum Quotient: Children’s Version (AQ: Child; Auyeung, Baron-Cohen, Wheelwright and Allison, 2008)

Given the high correlation between anxiety and autistic traits, the AQ: Child was used to assess autism symptoms. The AQ: Child is a 50-item parent-report measure of autistic traits with good psychometric properties [43]. Parents were asked to rate each item indicating to what extent they agree or disagree with the statements about their child. The higher the score, the more autistic-like traits the child shows. In this study, the full scale showed good internal consistency (α = 0.83).

### 2.3. Measures: Children

#### 2.3.1. The Wechsler Preschool and Primary Scale of Intelligence (WPPSI-IV)

Wechsler Preschool and Primary Scale of Intelligence (WPPSI-IV) is an individually administered standardised test of cognitive development for children aged 2 years 6 months to 7 years 7 months. The WPPSI is a highly reliable and valid measure of general intelligence in children [44]. The individual scales, rather than the full test, of the WPPSI have been previously used for research purposes (e.g., [45]). In this study, children completed the verbal comprehension subscale as an assessment of verbal abilities and block design subscale as an assessment of nonverbal abilities. The measure includes current and developmentally appropriate norms against which individual child’s scores were measured and these norms have shown good reliability and validity [46]. Age equivalence was used as a metric of verbal and nonverbal cognitive abilities. To facilitate comparison between participants, we chose to use the WPPSI-IV with the entire sample, despite some being aged above 7 years 7 months. Eight participants were aged above 7 years 7 months and scored at the highest age equivalence. This means that their scores may be slightly underestimated.

#### 2.3.2. Attention Bias Task

Stimuli. Children were presented with a series of pictures of faces and objects displayed on a computer screen. Faces of children displaying anger, happiness and ‘neutral‘ mood were selected from the Radboud standardised set [47]. Six models (three male) were used, each presenting each emotion, thus resulting in a set of 24 faces. The faces were grey-scaled, and all features extraneous to the face were removed using Photoshop. Six neutral nonemotional pictures of objects from the International Affective Picture System (IAPS; [48]), matched on ratings of arousal, were also grey-scaled for use. Average luminosity across faces and objects was equal.

**Figure 1:**
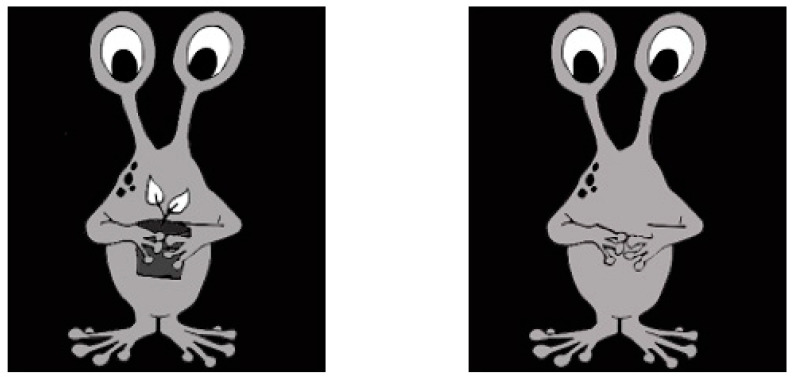
Example of alien stimuli used in the irrelevant task. In this example, the alien is seen holding a plant pot or not holding a plant pot.

Cartoon pictures of aliens were designed for the task. Three pairs of aliens were produced that differed in one characteristic per pair. These characteristics were: holding a plant or not holding a plant (see Figure 1); eye open or eye closed; upside down or the right way up. Average luminosity was equal across the alien images. The body of the alien was consistent across the aliens and only the eyes were present so no facial expression, and therefore emotion, could be interpreted from the alien’s facial features.

**Figure 2:**
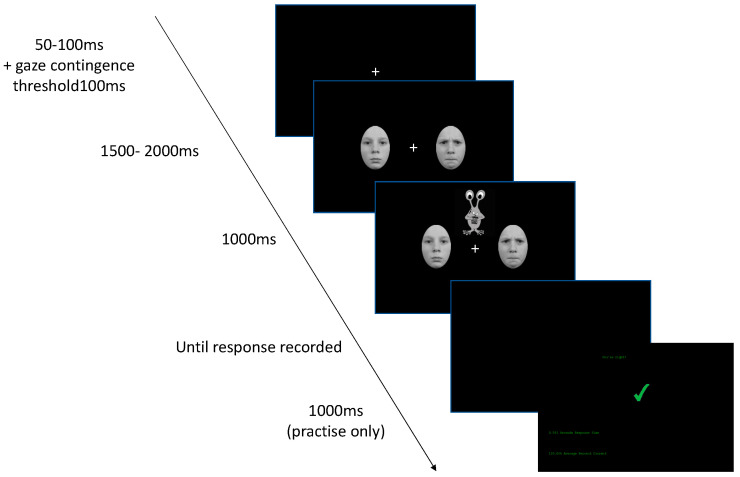
Schematic representation of practice and experimental trials.

Design. The task comprised a practice block and six experimental blocks. The practice block consisted of six trials. Each experimental block included 12 trials followed by a self-timed break. A 5-point eye-tracking calibration procedure was conducted before the practice block and before each experimental block. The procedure for practice and experimental trials is shown in Figure 2.

Each practice trial started with the presentation of a fixation cross. The initial presentation time of the cross was jittered (randomly selected) between 50 and 100 ms, and the cross remained on the screen until the children had fixated upon it for 100 ms. Two faces or nonemotional images then appeared to the right and left of the fixation cross. These images were presented for between 1500 ms and 2000 ms; the timing was randomised across trials and blocks. With the faces/nonemotional images still on the screen, an alien appeared above or below the centre of the screen. Participants were asked to categorise the alien verbally. Depending on the block, the task was to state whether the alien was holding a plant or not, whether the alien had its eye open or not and whether the alien was upside down or not. This task design meant that the faces were not relevant to the child’s task. Once the aliens had been displayed for 1000 ms, a blank screen appeared until the child’s verbal response to the alien was recorded. Once the experimenter recorded the response with a mouse click, feedback appeared on the screen for 1000 ms. On-screen feedback informed the children either “You’re right” and a green tick or “Ooops” with a red cross. The aliens were included to engage the children and keep them actively attending to the task. No data were analysed for the section of the trials when the alien was on the screen. The verbal responses allowed the experimenter to monitor the child’s level of attention and engagement with the task and provide encouragement if needed. Performance on the secondary task did not influence the selection of trials or participants for the analyses.

The experimental blocks of 72 trials involved presenting pairs of either nonemotional pictures (12 trials) or emotional faces (24 angry–neutral, 24 happy–neutral, 12 neutral–neutral). The experimental trials were identical to the practice trials, except no feedback was given. Trial type was randomised within experimental blocks. One of the three alien pairs was randomly assigned to each experimental block and the alien presented on each trial was randomised. Face pairs were randomised such that the same number of emotion–neutral and neutral–neutral pairings were seen in each experimental block and that the actors were seen the same number of times over the task.

### 2.4. Procedure

Study procedures were approved by the University of Reading Ethics Committee (Ref: 2014-025-HD). Participants attended a research session on campus during which the eye-tracking (attention bias) task and questionnaires outlined above were completed. During the same session, participants also completed an interpretation bias task, the findings of which are reported separately for clarity and due to space limitations [49]. In addition, during the session, we collected the following data, which we have no plans to publish: maternal anxiety; interpretation of ambiguous scenarios (for 7- and 8-year-olds only).

When families arrived for the session, parents provided informed consent for their child and verbal assent was obtained from the child. Parents completed the parent-report questionnaire measures while the child completed the experimental tasks. Generally, children completed the eye-tracking task, the WPPSI subtasks and then the interpretation bias task. On completion of all the tasks, the parent was given a debrief sheet and £5 towards travel expenses. All children received a certificate, stickers and a token prize for their co-operation and time.

For the eye-tracking task, children were introduced to the eye-tracking computer, the 5-point eye-tracking calibration procedure was conducted and the practice block was introduced. Children were told they would first see a cross, then some faces or pictures of objects would appear on the screen and then an alien would appear at the top or bottom of the screen. Children were told that once the alien appeared, they should say if the alien was, for example, upside down or the right way up. Once the practice block was completed the first experimental block was introduced. Children were informed when they were halfway through the experimental blocks and their willingness to continue was checked. Once the child had completed all six experimental blocks or refused to continue, the task ended.

### 2.5. Recording and Preprocessing of Eye-Tracking Data

Eye-tracking data were recorded using a Tobii T60, stimuli were presented via E-prime version 2.0 [50] and data were recorded through the Tobii extension for E-prime [51]. Children, on average, sat 55 cm from a 17-inch monitor. The centre of the IAPS and Radbound face images were placed to the right and left (4.5°) from the centre of the screen, with the inner edge of the image being 2° from the centre of the screen and the image subtended a visual angle of 4.9°. The cartoon alien images were 7.0° above and below the centre of the screen, with the inner edge being 3.5° from the centre of the screen and the alien images subtended a visual angle of 7.7°. Images were therefore placed within the parafoveal vision of the children.

Eye-tracking data were preprocessed using MATLAB version R2014b [52], R version 3.4.1(R Core Team, 2017) and R studio version 0.98.1103 [53] using gdata [54]. To assess whether eye-tracking data were valid, first, observation plots were checked to confirm that calibration was successful at the block, and if necessary, the trial level. Any trial or complete block that showed a systematic shift in eye-tracking coordinates indicating poor calibration was removed from the dataset. Next, for a trial to be valid, 5 consecutive samples out of the 10 observations (160 ms) before the onset of the faces/images had to be within 2° of the centre. This indicated that the child was looking at the centre when the faces/objects appeared on the screen. Any trials deemed “not valid” by this phase were excluded.

To prepare the data for GCA, data deemed valid by the previous checks were passed through the preprocessing functions of R package EyetrackingR [55]. This package assessed whether the eye tracker had registered the participant’s gaze per eye-tracking sample, if it had not, the sample was deemed invalid. If a trial contained 40% or more invalid observations it was excluded. Analyses were performed using R packages EyetrackingR [55] and lme4 [56] as well as ggplot [57], matrix [58] and tidyverse [59].

### 2.6. Statistical Analysis

Initially, we examined differences between children with high and low anxiety on potential confounding variables, e.g., autistic quotient or gender. Any variables that showed a potential confound and were not already in the analysis plan were included in further analyses. To evaluate the research hypotheses, two approaches were taken. First, conventional repeated ANOVAs were conducted on summary statistics derived from the eye-tracking data following preprocessing. GCA was then used to capitalise on the continuous nature of eye-tracking data to build upon the summary statistic results. The details of each approach are provided within the relevant section of results below, and further details can be found in the Supplementary Materials.

## 3. Results

### 3.1. Differences Between High- and Low-Anxious Groups

There were no age differences between children with high and low anxiety (high-anxious group: *M* = 6.04, *SD* = 1.15; low-anxious group *M* = 5.99, *SD* = 1.22; *t*(80) = 0.172 *p* = 0.863) or gender ($X^{2}$(1) = 0.096 *p* = 0.76). High- and low-anxiety groups were found to differ on autistic quotient score where, on average, the high-anxious group (*M* = 63.27, *SD* = 16.97) showed more autistic traits than the low-anxious group (*M* = 54.62, *SD* = 15.38) with a large effect size (*t*(99) = 4.43, *p* < 0.0001, *d* = 0.89). Autistic quotient scores were therefore included in further analyses as a covariate. Groups also differed on total effortful control score (Mann–Whitney *U* = 1603.5, *p* = 0.02), where the high-anxious group (*M* = 4.38, *SD* = 1.14) had lower scores than the low-anxious group (*M* = 4.91, *SD* = 0.68). Effortful control was not used as a covariate because the effect of effortful control was already included in the planned analysis. There were no differences between anxiety groups on nonverbal cognitive abilities (high-anxious group: *M* = 5.56, *SD* = 1.30; low-anxious group: *M* = 5.56, *SD* = 1.30) or verbal cognitive abilities (high-anxious group: *M* = 5.73, *SD* = 1.25; low-anxious group: *M* = 5.93, *SD* = 1.32).

### 3.2. Repeated-Measures Analysis

Two dependent variables (DV) were calculated for each child for use as summary statistics in mixed ANCOVAs. ‘Initial vigilance‘ was calculated as the proportion of trials in which each child looked at the emotional face before the neutral face. ‘Length of first look’ was calculated as the average length of the first look at a face as a proportion of the total time the child looked at faces across the trial. Three mixed ANCOVAs were run on each DV. The first examined anxiety differences and included one within-participant factor (emotion; happy/angry), one between-participant factor (anxiety group; high/low) and autistic quotient scores were entered as a covariate. The second examined age as a moderator, thus added age as a between-participant factor to the first model. The main effects and interactions with age were included in this second model. The third ANCOVA examined the influence of effortful control on the age-related effects, thus effortful control was added as between-participant factors to the moderation model. For brevity, only results pertaining to the research questions will be described, although full results can be found in Appendix A.

#### 3.2.1. Proportion of Trials Where the Initial Look to Faces Was to the Emotional Face

Table 1 shows the average proportion of trials where the initial look was to the emotional face in the angry and happy trials by anxiety groups. The means in Table 1 suggest that children initially looked at the emotional face in around 60% of the trials. This was significantly more often than chance (50%) for angry faces (*t*(101) = 3.92, *p* < 0.001, *d* = 0.78) and for happy faces (*t*(99) = 6.32, *p* < 0.001, *d* = 1.27), suggesting that children were vigilant to both emotional faces. The mixed ANCOVA assessing anxiety differences revealed there were no differences between happy or angry trials on this DV and, importantly, no differences between children with high or low anxiety and no significant two-way interaction between emotion type and anxiety group (all *p*’s > 0.2 for main effects and interactions). There were also no significant interactions between child anxiety and age or main effect of anxiety in the moderation by age model (all *p*’s > 0.09 for main effects and interactions), nor was there a main effect of effortful control when it was added to the moderation by age model in the third ANCOVA. Results from the ANCOVAs therefore indicate that there was no difference in vigilance to the emotional faces by emotion or by anxiety grouping, nor was any influence by age or effortful control on the proportion of trials the children initially looked to the emotional face when controlling for autistic traits.

#### 3.2.2. Proportion of Time Spent Looking at the Faces During Initial Looks

Figure 1 also shows the average proportion of time anxious and low-anxious children spent looking at each happy, angry or neutral face. Mixed ANCOVA analysis investigating anxiety differences showed no main effects of emotion type, face type or anxiety level and no significant interactions between emotion type and anxiety grouping. In the moderation by age analysis, there were main effects of age (*F*(40) = 2.77, *p* = 0.007, $\eta_{p}^{2}\;$= 0.84) and emotion type (*F*(40) = 13.45, *p* = 0.001, $\eta_{p}^{2}\;$= 0.39). Furthermore, there was a significant interaction between age, face type and emotion type (*F*(40) = 2.64, *p* = 0.01, $\eta_{p}^{2}\;$= 0.83). To investigate this interaction, the data were split into younger (below mean age) and older (above mean age) children, and the analysis was re-run. The interaction between emotion type and face type only remained for the younger children (*F*(1) = 4.45, *p* = 0.04, $\eta_{p}^{2}\;$= 0.09). The interaction indicated that, relative to happy trials, young children looked for a smaller proportion of time at the emotional face on angry trials (*M* = 0.36, *SD* = 0.18) and for a greater proportion of time at the neutral face (*M* = 0.16, *SD* = 0.16) (happy trials: *M_emotional_* = 0.44, *SD_emotional_* = 0.20; *M_neutral_* = 0.13, *SD_neutral_* = 0.14). These results may suggest that in terms of initial looks, young children avoided the angry face by looking at it for less time before moving attention away. However, this pattern of attention was not affected by anxiety levels. In the final ANCOVA model, adding effortful control to the moderation by age model did not substantially alter the results and the main effect of effortful control was not significant (see Appendix A for full results).

### 3.3. Growth Curve Analyses: Initial Looks to Faces

#### 3.3.1. Data Preparation

Growth curve analysis was conducted on eye-tracking data recorded while the faces were on the screen (up to 2000 ms) using data from the first look to the first face. Analysis was performed following the procedures of Mirman et al. [36] as well as examples from Byrow, Chen and Peters [37] and Schofield, Inhoff and Coles [60]. The dependent variable was biased in the proportion of time spent looking at an emotional face (proportion of observations per time bin on the emotional face minus the proportion of observations per time bin on the neutral face). Further detail regarding data preparation and statistical analysis can be found in the Supplementary Materials.

GCA was used to investigate whether there were differences between children with high and low levels of anxiety in the time course of their gaze patterns. Random intercepts and slopes were modelled. Four models were created to investigate the hypotheses. The first (Model 1) investigated whether there were anxiety-related differences in initial looks to the faces using bias scores by emotion type (happy or angry). The fixed effects of group (high- and low-anxious children; between-subjects’ variable), emotion type and time variables and their interactions were entered in Model 1. Model 2 investigated moderation effects of age, thus the main effects of age and interaction with age were entered alongside Model 1 predictors. Model 3 investigated whether the age-related effects remained after controlling for effortful control by adding effortful control to Model 2. Finally, in Model 4, cognitive and linguistic abilities were entered as main effects to Model 1. Autistic quotient was added as a predictor into all models as a covariate.

**Figure 3:**
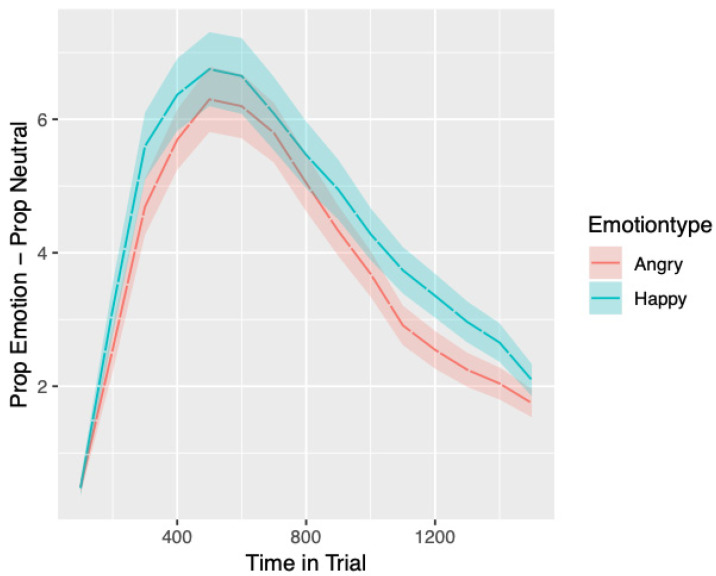
Time course of bias to emotional faces averaged over trials, split by emotion type. Standard errors in the proportion of looking to Areas of Interest (AOIs) can be seen as shaded areas surrounding the lines denoting emotion type.

After preprocessing and preparation for analysis, 27,177 samples from 104 children were included in the analysis. Given the number of models, model parameters and the number of significant parameters found, focus is given here to results that pertain to the hypothesised main effects of emotion type, differences in the time course of visual attention influenced by anxiety (interactions with anxiety), moderation by age (interactions between age and anxiety) and the influence of effortful control (main effect), verbal and nonverbal cognitive abilities (main effects). For brevity, only significant main effects and interactions described above will be fully reported. The time course of bias scores by emotion type can be seen in Figure 3.

#### 3.3.2. Model 1: Influence of Anxiety Level on Attention Bias

**Figure 4:**
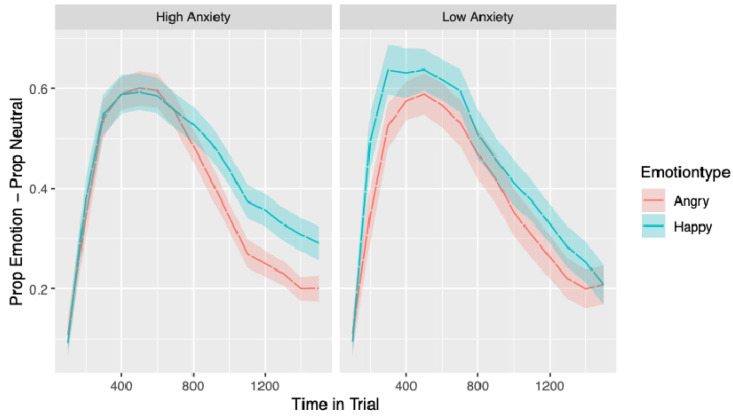
Time course of bias to looking at the emotional face as the first face viewed averaged over trials, split by emotion type and anxiety grouping. Standard errors in the proportion of looking to AOIs can be seen as shaded areas surrounding the lines denoting anxiety groups.

The time course of bias scores by emotion and anxiety can be seen in Figure 4. The main effect of emotion indicates that on average children were more likely to look initially to an emotional face than a neutral face (*b* = −0.07, *SE* = 0.02 *t* = −5.55, *p* < 0.001). There were also main effects linear time (*b* = −0.87, *SE* = 0.06, *t* = −14.78, *p* < 0.001) and quadratic time (*b* = −0.80, *SE* = 0.05, *t* = −14.76, *p* < 0.001), indicating that looking behaviour to faces changed over time. No other main effect or interactions were significant. However, the interaction between emotion, anxiety and linear time was close to significance (*b* = −0.23, *SE* = 0.13, *t* = −1.82, *p* = 0.069). This result, in conjunction with Figure 4, is suggestive of a steeper decline over the trial in bias in the angry trials relative to the happy trials for the high-anxious group only.

#### 3.3.3. Model 2: Moderation of the Relationship Between Anxiety and Attention Bias by Age

Compared to the model assessing differences in gaze between anxious and low-anxious children, adding age improved the model fit ($\chi^{2}$ (20) = 58.35, *p* < 0.05) and explained some residual variance (3.13%).

**Figure 5:**
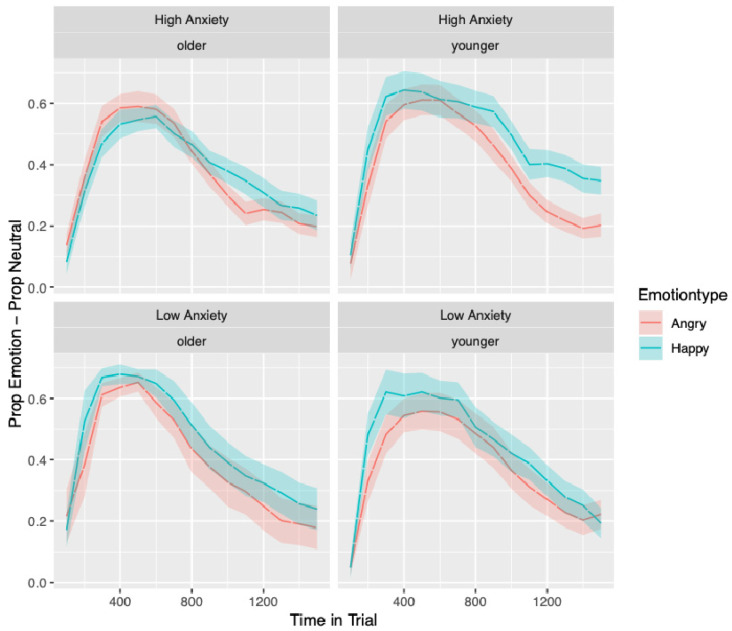
Time course of bias to looking at the emotional face as the first face viewed averaged over trials, split by emotion type, age and anxiety grouping. Standard errors in the proportion of looking to AOIs can be seen as shaded areas surrounding the lines denoting anxiety groups.

There was one significant three-way interaction between emotion type, anxiety and age (*b* = 0.08 *SE* = 0.02 *t* = 3.48, *p* < 0.001). No other interactions reached significance. However, a number of other interactions approached significance: the three-way interaction between emotion type, anxiety and linear time (*b* = −0.22 *SE* = 0.13 *t* = −1.77, *p* = 0.078); emotion type, age and quadratic time (*b* = 0.11, *SE* = 0.06 *t* = 1.88, *p* = 0.06); emotion type, anxiety, age and linear (*b* = 0.18 *SE* = 0.11 *t* = 1.66, *p* = 0.098) and quadratic time (*b* = 0.19 *SE* = 0.12 *t* = 1.65, *p* = 0.099). These are relevant given that including age significantly improved the model. These results, in combination with Figure 5, which visualises the four-way interaction, suggest that if there are any age-related anxiety effects, they are being driven by the younger children in the high-anxious group. For all other groups, the decline in bias between the emotions from the beginning to the end of the trial runs relatively parallel, but for the younger anxious group, the decline in bias across the trial appears to be steeper in the angry trials compared to the happy trial. This difference likely underpins the significant emotion, anxiety and age interaction, as young, high-anxious children show a difference in gaze to angry relative to happy that is not seen in other groups. Figure 5 also suggests that older children in the high-anxious group show the reverse pattern to all other groups in initial bias: older children in the high-anxious group show a larger initial bias to the *angry* face than the happy face, and all other groups show a larger initial bias to looking at the *happy* face than the angry face.

#### 3.3.4. Model 3: Do Age Effects Remain When Controlling for Effortful Control?

Model fit was improved relative to Model 2 ($\chi^{2}$(32) = 58.13, *p* < 0.05) and explained some variance in the subject level (7.23%). Despite this, there were no main effects of effortful control and the results were very consistent with those for Model 2 (see above), suggesting that when the common variance between age and effortful control was removed from the model, age effects remained. Thus, age effects cannot be accounted for by effortful control.

#### 3.3.5. Model 4: Influence of Verbal and Nonverbal Cognitive Abilities on Initial Looks to Faces

The results were similar to those found in examining anxiety differences (see Model 1). Model fit was not improved over Model 1 ($\chi^{2}$(20) = 0.89, *p* > 0.05) and explained a small amount of variance on the level of the subject (1.35%). There were no main effects of verbal or nonverbal cognitive abilities, suggesting they did not influence initial looking behaviour to the faces.

## 4. Discussion

This paper aimed to establish whether children aged four to eight years show an anxiety-related attention bias to emotional stimuli. It was hypothesised that all children would show an initial vigilance to emotional faces, irrespective of anxiety level. Results from conventional analyses (*t*-tests and ANCOVAs) supported this hypothesis, indicating that children were vigilant to both angry and happy faces relative to neutral. Support for this hypothesis can also be seen in the growth curve (GCA) models where there is a steeper rise in the proportion of looking to emotional faces relative to neutral faces within the first 500 ms of trials. It was further hypothesised that this initial vigilance would be stronger for angry than for happy faces, again irrespective of anxiety. However, neither the conventional nor the GCA analyses supported this hypothesis. Overall, therefore, the results indicate that children aged four to eight in general show a bias for emotional faces over neutral faces.

In relation to anxiety, we hypothesised that participants with higher levels of anxiety would show increased vigilance for angry faces followed by avoidance, relative to participants with lower levels of anxiety. Taking vigilance first, neither the results from the conventional analyses nor the GCA models suggested differences in vigilance between anxiety groups. However, it should be noted that the results of the moderation by age analysis hinted that older children in the high-anxious group may have a larger initial bias to angry faces than happy, which was not observed in any other group. The lack of evidence for an anxiety-linked vigilance bias for angry faces is inconsistent with much of the adult literature showing a bias [5]. It is important to note though that more recent work has failed to find an anxiety-linked bias even in adults [61].

We hypothesised that the strength of any anxiety-related vigilance bias would be moderated by age. This was based on previous work that found differences in anxiety-related attention bias by age [18,19] as well as a recent meta-analysis of the anxiety–bias relationship [6]. There was no support for this hypothesis in relation to vigilance for angry faces. There are a number of possible explanations for this. It may be due to the present sample being younger than the samples used in previous research finding moderation by age. Alternatively, the difference in results may be due to different methodologies (free-viewing versus dot-probe tasks) or differences in the nature of the sample (clinical vs. community). Regardless, the results are broadly consistent with moderation by age effect when taken together with the wider literature. The vigilance for emotional faces over neutral faces, irrespective of anxiety, aligns with previous research on attention bias in young children [34] and the broader developmental literature on children’s attention to emotional faces [62,63,64]. Within studies of attention bias and anxiety in older children (aged 8+ years) a general bias towards emotional stimuli (angry and happy) over neutral has also been found but this bias is often stronger in more anxious children [65,66,67]. Taken together with our findings, this supports the idea that a general vigilance for emotional faces is present early in childhood, but anxiety-related vigilance only emerges as children get older. This would be in keeping with the moderation model of Field and Lester [17] as well as the Inhibition Hypothesis [26].

Although we did not find the anticipated anxiety-related differences in *vigilance*, there was evidence for anxiety-related differences in *avoidance*. The interaction between anxiety group, emotion and linear time seen in Model 1 approached significance and suggested that children in the high-anxious group looked away from angry faces faster than happy faces and faster than children in the low-anxious group. In Model 2, this effect appeared to be moderated somewhat by age as it was only apparent for the younger children (see Figure 5). This means that the strongest evidence for moderation by age effects within our data is for avoidance and are driven by the young children in the high-anxious group avoiding angry faces relative to the happy faces. This is the reverse of what we had anticipated and what would be expected from theories of attention where withdrawal of attention or avoidance is thought to be dependent on top-down control [25], which develops across childhood [22]. Given that the younger children cannot have better top-down control than the older children, their withdrawal of attention must be driven by a relatively automatic process that leads young high-anxious children to move their attention away from threat once it is detected. This interaction effect did not reach statistical significance and these effects were not hypothesised; therefore, we strongly recommend the replication of these findings in future research.

As theory and previous research suggest that effortful control may be a relevant developmental factor in the relationship between attention bias and anxiety, it was investigated whether any age-related effects remained after controlling for effortful control. Adding effortful control improved model fit over the moderation by age model and explained some variance on the subject level. However, the results of the moderation by age model remained unchanged when effortful control was added as a main effect and effortful control was not a significant predictor. These results indicate that age-related effects suggested by the moderation by age model were not driven by effortful control.

There was no evidence of any influence of verbal and nonverbal cognitive abilities on task performance. Neither model fit indices showed improvement over the model investigating anxiety differences nor were verbal and nonverbal cognitive abilities significant predictors of bias scores. This is important to note as previous tasks used with young children have been criticised as performance on these tasks was dependent on verbal and nonverbal cognitive abilities. Our results suggest that such criticisms would not apply to this task and that the task was appropriate to the developmental level of the sample in terms of verbal and nonverbal cognitive abilities. Previous tasks used to assess attention biases in children, i.e., the dot-probe, have been criticised for their complex instructions and reliance on developing abilities. By avoiding the use of reaction times and utilising a free-viewing paradigm within an eye-tracking task we have shown it is possible to create a task that is not duly influenced by verbal and nonverbal cognitive abilities.

### 4.1. Implications for Treatment

We found little evidence for an anxiety-linked attention bias for threat in young children. This suggests that Attention Bias Modification (ABM) approaches to changing bias used with older children and adults, e.g., [68,69] are unlikely to be appropriate in early childhood. Given that participants were recruited from the community, it remains possible that an anxiety-linked bias would be observed in clinically diagnosed young children. This seems unlikely given that Dodd and colleagues [34] did not find an anxiety-linked bias in their sample of diagnosed three- and four-year-olds, but replication with a clinical sample would be beneficial.

### 4.2. Strengths and Limitations

This study made use of eye tracking to assess whether an anxiety-related attention bias is present in children aged four to eight, an age group neglected by the literature until now. This adds to the growing body of evidence using eye tracking to assess attention bias in children. One of the strengths of the study is the use of GCA. Using this technique allowed for a fine-grained analysis of overt attention to emotional stimuli that reaction time data and conventional assessment of eye-tracking data cannot provide. The GCA revealed particular patterns that were not apparent in the conventional analyses, such as the withdrawal of attention from faces in young, high-anxious children.

A further strength is the consideration of developmental factors that may influence task performance and moderate the associations of interest. We demonstrated that task performance was relatively unaffected by children’s verbal and nonverbal cognitive abilities. In addition, the results suggest that development (age) may influence the anxiety–attention bias relationship. Until now, it has been rare for the impact of developmental factors on the relationship between anxiety and attention biases to be examined. Although several studies have assessed the influence of age, very few have assessed the influence of effortful control despite it being acknowledged as being theoretically relevant for the attention bias–anxiety relationship [17].

There are limitations concerning the sample. As participants were recruited via adverts, the sample is self-selecting, and the study focused on recruitment in Berkshire, an area predominantly made up of white British families [70], which is reflected in our sample. Both of these factors limit the extent to which the results can be generalised. After experiencing problems recruiting very-low-anxious children, we adjusted the eligibility criteria for the low-anxious group to those with anxiety scores below the normed mean. This resulted in a smaller difference in anxiety between groups than originally intended. As discussed above, it may be that future studies using a sample made up of clinically anxious children versus healthy controls may find stronger effects of anxiety. Furthermore, the absence of significant findings, but close-to-significance findings may be due to a lack of power of the study. Although the sample was powered to look at anxiety effects, models also included random effects, random slopes and a lot of interactions with time, which would have limited the power of the study to detect significant multiway interactions. Future studies with children aged four to eight years with greater power should be conducted to confirm the nature of anxiety-related biases.

A further limitation is that the influence of development was assessed within a cross-sectional design. To really understand the role of development, as well as the role of cognitive biases in the development of anxiety, longitudinal work is required. To get a clearer picture of the nature of bias and how or if this changes over time, eye-tracking methods analysed with growth curve models would be employed over different developmental time points. In this study, a Tobi60 eye tracker was used to collect data. However, eye trackers with a much higher sampling rate are now available and suitable for use with children, which may also allow greater precision in mapping the time course in visual attention and elucidating differences.

The lack of results concerning the influence of effortful control may also be due to the measure of effortful control being a parent questionnaire rather than a direct performance-based measure. Effortful control reflects a set of skills that children use to control their behaviour; however, the parent measure reflects parents’ perception of a particular set of behaviours derived from observations in order to infer children’s effortful control abilities. Performance-based measures of attention control and inhibition may provide a more direct measure of effortful control. For example, the antisaccade task [71,72] could be adapted for a young age group and used to assess the influence of effortful control on the relationship between attention bias and anxiety in young children. In this study, only the influence of effortful control on moderation by age effects was assessed; however, ideally, effortful control would have been assessed as a moderator. As the measure was restricted to a parent report, moderation was not assessed; however, should a performance-based measure be used, moderation by effortful control could be investigated. Future research may also look at other potential moderators including social and emotional factors, not considered here, which may explain changes with age. For example, previous research has considered the impact of attachment style on the relationship between social anxiety and attentional biases [37]. Socioemotional factors such as emotion regulation or facial emotion recognition may also be important [17].

## 5. Conclusions

This study used a novel, child-friendly eye-tracking task to assess if there is evidence of an anxiety-related attention bias to threat in children aged four to eight. There was little evidence of anxiety differences in initial looks to faces; all children showed vigilance for emotional faces over neutral faces. Analysis of moderation by age revealed nuanced differences in visual attention to faces between anxiety groups across age. Specifically, there was some indication of more pronounced differences between anxiety groups in the younger children. The results are broadly consistent with a moderation model. Future studies should extend this work to a clinical sample and use a longitudinal design to capture the nature of the role of developmental factors in the anxiety–attention bias relationship in young children.

## Figures and Tables

**Table 1:** Summary statistics of initial looks to the faces by anxiety group.

	Mean Proportions (SD)		
	Total	High-Anxious Group (*n* = 65)	Low-Anxious Group (*n* = 39)
Mean Proportions of Trials Where the Initial Look Was to the Emotional Face			
Angry Trials	0.59 (0.22)	0.60 (0.23)	0.57 (0.20)
Happy Trials	0.63 (0.21)	0.64 (0.22)	0.63 (0.20)
Mean Proportions of Time Spent Looking at Faces During Initial Looks			
Angry Trials			
Emotional Face	0.35 (0.17)	0.35 (0.16)	0.36 (0.18)
Neutral Face	0.16 (0.16)	0.15 (0.15)	0.17 (0.16)
Happy Trials			
Emotional Face	0.40 (0.19)	0.41 (0.20)	0.39 (0.20)
Neutral Face	0.13 (0.12)	0.13 (0.13)	0.13 (0.11)

## Supplementary Materials

The following are available online at https://www.mdpi.com/2076-328X/10/12/194/s1, Technical Supplement.

## Appendix A

**Table A1:** Results of the mixed ANCOVAs for mean proportions of trials where the initial look was to the emotional face.

	df	*F*	$\eta_{p}^{2}$	*p*
**Anxiety Differences**				
Anxiety	1	0.002	0	0.968
Emotion type	1	0.002	0	0.966
Autistic Quotient	1	1.56	0.02	0.215
Anxiety × Emotion type	1	0.001	0	0.999
**Moderation by Age**				
Anxiety	1	1.01	0.05	0.325
Autistic Quotient	1	2.5	0.11	0.128
Age	40	1.72	0.77	0.091
Anxiety × Emotion type	1	1.59	0.07	0.221
Anxiety × Age	10	0.35	0.14	0.956
Emotion type × Age	40	1.79	0.77	0.078
Anxiety × Age × Emotion type	10	0.20	0.09	0.993
**Influence by Effortful Control**				
Anxiety	1	0.49	0.002	0.829
Emotion type	1	2.04	0.17	0.183
Autistic Quotient	1	1.85	0.16	0.160
Effortful Control	1	3.15	0.14	0.091
Age	40	1.85	0.79	0.071
Anxiety × Emotion type	1	1.46	0.07	0.241
Anxiety × Age	10	0.54	0.21	0.845
Emotion type × Age	40	1.70	0.77	0.101
Anxiety × Age × Emotion type	10	0.20	0.09	0.994

Note. Although interactions with autistic quotients were included, they are not in the table for brevity.

**Table A2:** Results of the mixed ANCOVAs for mean proportions of time spent looking at faces during Initial Looks.

	df	*F*	$\eta_{p}^{2}$	*p*
**Anxiety Differences**				
Anxiety	1	0.09	0.001	0.768
Emotion type	1	1.85	0	0.966
Face Type	1	2.03	0.03	0.158
Autistic Quotient	1	0.74	0.01	0.394
Anxiety × Emotion type	1	0.19	0.003	0.668
Anxiety × Face type	1	0.03	0	0.873
Emotion type × Face type	1	1.16	0.02	0.285
**Moderation by Age**				
Anxiety	1	1.32	0.06	0.262
Emotion type	1	13.45	0.39	0.001 ***
Face type	1	0.95	0.04	00.341
Autistic Quotient	1	0.001	0.986	0.001
Age	40	2.77	0.84	0.007 **
Anxiety × Emotion type	1	0.95	0.04	0.341
Anxiety × Age	10	0.66	0.24	0.746
Anxiety × Face type	1	0.21	0.01	0.655
Emotion type × Age	40	1.65	0.78	0.341
Emotion type × Face type				
Age × Face type	40	2.64	0.83	0.01 **
Anxiety × Age × Emotion type	10	2.06	0.50	0.078
Anxiety × Age × Face type	10	0.40	0.16	0.933
Age x Emotion type × Face type	40	2.10	0.80	0.039 *
Anxiety × Age × Emotion type × Face type	10	0.57	0.21	0.822
**Influence by Effortful Control**				
Anxiety	1	1.35	0.06	0.257
Emotion type	1	2.36	0.10	0.139
Face type	1	0.04	0.002	0.840
Autistic Quotient	1	1.40	0.06	0.250
Effortful control	1	1.79	0.08	0.194
Age	41	2.26	0.81	0.022 *
Anxiety × Emotion type	1	0.68	0.03	0.419
Anxiety × Age	11	1.08	0.35	0.421
Anxiety × Face type	1	0.16	0.01	0.695
Emotion type × Age	41	2.68	0.83	0.008 *
Emotion type × Face type	1	1.33	0.06	0.260
Age × Face type	41	1.94	0.78	0.049 *
Anxiety × Age × Emotion type	11	1.36	0.41	0.259
Anxiety × Age × Face type	11	0.58	0.22	0.828
Anxiety × Emotion type × Face type	1	0.64	0.03	0.431
Age × Emotion type × Face type	41	0.95	0.64	0.566
Anxiety × Age × Emotion type × Face type	11	0.16	0.07	0.998

Note. * *p* < 0.05; ** *p* < 0.01.
