# Supplementary material for: Anxiety-Related Attention Bias in Four- to Eight-Year-Olds: An Eye-Tracking Study
Source: Behav Sci (Basel). 2020 Dec 17;10(12):194. doi: 10.3390/bs10120194 (PMC7766356; doi:10.3390/bs10120194)
Supplement: Supplementary file 1 [file behavsci-10-00194-s001.pdf]

Technical Supplement

Data Preparation

Growth curve analysis was conducted on eye-tracking data that was recorded while the faces were on screen (2000ms). Time was split into 100ms time bins and the dependent variable, bias in the proportion of time spent looking at an emotional face (proportion of observations per time bin on the emotional face minus the proportion of observations per time bin on the neutral face), was calculated per time bin. To ensure the same behavioural event for each participant was assessed, initial looks to each AOI were computed in the following way. The proportion was calculated whereby each eye-tracking sample showing a valid observation within a time bin was allocated 1 if its location was within a face AOI and 0 if it was not. The allocations were summed and then divided by the number of samples within that time bin to give proportion of samples within AOI. Proportions for each time bin were then averaged over trial type (happy vs neutral, angry vs neutral), then participants. Raw proportions were transformed using a logit adjustment, which was used as the dependent variable. The bounded nature of proportions can cause issues in analyses, the transformation is used to assist in resolving this issue. To make sure that we did not include anticipatory eye movements we did not include the first 100ms after each face was shown.

Statistical Analysis

GCA was used to investigate whether there were differences between anxious and non anxious children in the time course of their gaze patterns. For all GCA models the time course of the bias in the proportion of time spent looking at an emotional face was modelled using second-order (linear and quadratic) orthogonal polynomials (time codes). Linear effects reflect change in visual attention to faces over time. Quadratic effects were included in order to investigate whether there anxiety differences in vigilance/orienting to the faces and in avoidance as per the hypotheses. Fixed effects in the model were trial/emotion type (happy vs neutral, angry vs neutral; within subjects) and face type (emotional expression vs neutral expressions; within subjects). Four models were created to investigate the hypotheses. The first investigated whether there were anxiety differences in initial looks to the faces and whether this differed by whether the face was emotional or neutral or by emotion (happy or angry). Fixed effects of group (high and low anxious children; between subjects variable), and the interactions between emotion type, face type and anxiety group were entered in Model 1. Model 2 investigated moderation effects of age thus, fixed effects of age (between groups variable) and the interactions of age with emotion type, face type, anxiety and time

polynomials. Model 3 investigated whether the age related effects remained after controlling for effortful control. In Model 3 fixed effects of effortful control (between groups variable) was therefore added to Model 2. Finally, in Model 4 cognitive and linguistic abilities were entered as fixed effects (between groups variable) alongside emotion, anxiety grouping and time to assess whether they influenced visual attention to the faces. Continuous predictors were centred and dummy codes were produced for binary variables. Anxiety grouping, emotion type and face type were dummy coded such that the low anxious group, the happy trials and emotional faces were the respective reference categories. All GCA models used data from the first look to the first face and included random intercepts and slopes.
